# Heterogeneous changes in gut and tumor microbiota in patients with pancreatic cancer: insights from clinical evidence

**DOI:** 10.1186/s12885-024-12202-z

**Published:** 2024-04-15

**Authors:** Feng Zhao, Anli Chen, Xiaotian Wu, Xiangyu Deng, Jiali Yang, Jianjiang Xue

**Affiliations:** 1 Department of Health Laboratory Technology, School of Public Health, Chongqing Medical University, No. 55, Daxuecheng Middle Road, ShaPingBa District, 400016 Chongqing, People’s Republic of China; 2 Department of Clinical Laboratory, University-Town Hospital of Chongqing Medical University, 401331 Chongqing, People’s Republic of China; 3 Institute of Hepatopancreatobiliary Surgery, Chongqing General Hospital, 401147 Chongqing, People’s Republic of China

**Keywords:** Pancreatic cancer, Tumor microorganisms, Gut microbes, Tumor markers

## Abstract

**Background:**

Pancreatic cancer is the foremost contributor to cancer-related deaths globally, and its prevalence continues to rise annually. Nevertheless, the underlying mechanisms behind its development remain unclear and necessitate comprehensive investigation.

**Methods:**

In this study, a total of 29 fresh stool samples were collected from patients diagnosed with pancreatic cancer. The gut microbial data of healthy controls were obtained from the SRA database (SRA data number: SRP150089). Additionally, 28 serum samples and diseased tissues were collected from 14 patients with confirmed pancreatic cancer and 14 patients with chronic pancreatitis. Informed consent was obtained from both groups of patients. Microbial sequencing was performed using 16s rRNA.

**Results:**

The results showed that compared with healthy controls, the species abundance index of intestinal flora in patients with pancreatic cancer was increased (*P* < 0.05), and the number of beneficial bacteria at the genus level was reduced (*P* < 0.05). Compared with patients with chronic pancreatitis, the expression levels of CA242 and CA199 in the serum of patients with pancreatic cancer were increased (*P* < 0.05). The bacterial richness index of tumor microorganisms in patients with pancreatic cancer increased, while the diversity index decreased(*P* < 0.05). Furthermore, there was a change in the species composition at the genus level. Additionally, the expression level of CA242 was found to be significantly positively correlated with the relative abundance of Acinetobacter(*P* < 0.05).

**Conclusion:**

Over all, the expression levels of serum tumor markers CA242 and CA19-9 in patients with pancreatic cancer are increased, while the beneficial bacteria in the intestine and tumor microenvironment are reduced and pathogenic bacteria are increased. Acinetobacter is a specific bacterial genus highly expressed in pancreatic cancer tissue.

**Supplementary Information:**

The online version contains supplementary material available at 10.1186/s12885-024-12202-z.

## Background

Pancreatic cancer (PC) is a highly invasive and deadly disease, with a 5-year survival rate of less than 9% [1]. It is predicted that by 2040, pancreatic cancer will become the second leading cause of cancer deaths [2]. Due to the anatomical structure of pancreas, pancreatic cancer reaches advanced stage when patients have symptoms. Additionally, there is currently a lack of biomarkers for early diagnosis and accurate prognosis of pancreatic cancer.

The biomarker carbohydrate antigen 19 − 9 (CA19-9) is commonly employed in the diagnosis and prognosis of pancreatic cancer, it has a diagnostic sensitivity of 0.78 and a specificity of 0.77 [3]. However, patients with pancreatitis may exhibit reduced sensitivity to this test as a result of elevated CA19-9 concentrations. Furthermore, it should be noted that approximately 7-10% of the population may not demonstrate the presence of CA19-9 due to Lewis antigen deficiency [4]. Carbohydrate antigen 242 (CA242) is a carbohydrate antigen containing sialic acid, which can be found attached to core proteins or lipids on the cell surface or in serum [5]. Additional studies have revealed that CA242 has similar sensitivity and specificity to CA19-9 in the diagnosis of pancreatic cancer [6]. While the sensitivity of CA242 is generally low, it exhibits higher specificity in the diagnosis of pancreatic cancer. Notably, when CA242 is used in conjunction with CA19-9, both the sensitivity and specificity are significantly improved.

Microbiota can influence the development of precancerous disease predisposing to PC, at the same time, neoplastic tissue shows specific characteristics in terms of diversity and phenotype, determining the short-and long-term prognosis [7]. The search for biomarkers in gut microbiome research has generated significant interest. In 2018, the potential of the gut microbiome as a non-invasive diagnostic tool for hepatocellular carcinoma was discovered [8]. Furthermore, the gut microbiome currently provides promising biomarker research in lung, colon, and cervical cancers, as well as viral susceptibility [9]. Mendez and his team believe that in the early stages of pancreatic cancer, the dominant bacteria in the intestinal flora are Proteobacteria and Firmicutes [10]. On the other hand, studies have found that pathogenic bacteria and endotoxin-producing bacteria are increased in the intestinal flora of patients with pancreatic cancer, while probiotics and butyrate-producing bacteria are decreased [11]. These results suggest that gut microbiota composition may serve as a useful biomarker for early detection of pancreatic cancer.

The retrograde migration of intestinal microbiota through the pancreatic papilla to pancreatic tumors has been widely recognized [12]. Studies using mouse models have demonstrated that bacterial colonization in pancreatic cancer can reset immune tolerance and facilitate tumor progression through bacterial metabolites [13]. Interestingly, long-term survival in pancreatic cancer is associated with increased tumor bacterial diversity and numbers of mature CD8 + T cells and granular B cells, although the prognosis of this malignancy is often poor [14].

In this study, we collected stool and tissue from pancreatic cancer patients to examine changes in the microbiota between pancreatic cancer patients and matched controls. Statistical analysis methods were used to conduct correlation analysis on tumor markers in patients with pancreatic cancer and chronic pancreatitis. The purpose of this study was to find characteristic bacterial genera based on the microecology of pancreatic cancer.

## Material and method

### Study population

From 2020 to 2022, the Institute of Hepatopancreatic Surgery of Chongqing People’s Hospital collected stool samples from all 29 patients who were diagnosed with pancreatic cancer and underwent surgery and pathology. The individuals who were exposed to antibiotics, probiotics, and prebiotics 8 weeks before sampling, as well as those with a previous history of other cancers, acute or chronic intestinal inflammation, or known cancer-related mutations, were excluded from the analysis. Additionally, they were frozen in liquid nitrogen in a separate freezing bottle. From 2017 to 2022, pancreatic tumor tissue samples from 14 patients and 14 matching non-tumor (chronic pancreatitis) tissue samples were collected at the same institution. All samples were stored at -80 °C until further use. Download the 16 S rRNA V3-V4 data of the intestinal flora of the healthy control group from the SRA database (SRA accession number: SRP150089). This study was approved by the Ethics Committee of University Town Hospital of Chongqing Medical University (approval number: LL-202,022), and all patients signed informed consent forms.

### DNA extraction and PCR amplification

Total microbial genomic DNA was extracted from stool and tissue samples using the PF Mag-Bind Stool DNA Kit (Omega Bio-tek, Georgia, U.S.) according to manufacturer’s instructions. The quality and concentration of DNA were determined by 1.0% agarose gel electrophoresis and a NanoDrop® ND-2000 spectrophotometer (Thermo Scientific Inc., USA) and kept at -80 °C prior to further use. The hypervariable region V3-V4 of the bacterial 16 S rRNA gene were amplified with primer pairs 338 F (5’-ACTCCTACGGGAGGCAGCAG-3’) and 806R(5’-GGACTACHVGGGTWTCTAAT-3’) by an ABI GeneAmp® 9700 PCR thermocycler (ABI, CA, USA). The PCR reaction mixture including 4µL 5×Fast Pfu buffer, 2µL 2.5 mM dNTPs, 0.8µL each primer (5µM), 0.4µL Fast Pfu polymerase, 10 ng of template DNA, and ddH2O to a final volume of 20 µL. PCR amplification cycling conditions were as follows: initial denaturation at 95 °C for 3 min, followed by 27 cycles of denaturing at 95 °C for 30 s, annealing at 55 °C for 30 s and extension at 72 °C for 45 s, and single extension at 72 °C for 10 min, and end at 4 °C. All samples were amplified in triplicate. The PCR product was extracted from 2% agarose gel and purified. Then quantified using Quantus™ Fluorometer (Promega, USA).

### Illumina sequencing

Purified amplicons were pooled in equimolar amounts and paired-end sequenced on an Illumina MiSeq PE300 platform (Illumina, San Diego, USA) according to the standard protocols by Majorbio Bio-Pharm Technology Co. Ltd. (Shanghai, China).

### Microbiome Analysis

The obtained Raw Data was quality controlled using fastp software [15], reads were spliced using FLASH software [16], samples were differentiated according to barcode and primers, OTU clustering was performed using UPARSE [17]software, and finally the Silva 16 S rRNA gene database was annotated with OTU species taxonomy using RDP classifier than with a confidence threshold of 70% [18]. α diversity was calculated using mothur software [19]. The indices used to calculate bacterial community richness include the Chao index and Ace index, these indices are primarily used to estimate the total number of species in a community, a higher index value indicates a greater community richness. On the other hand, the Shannon index and Simpson index are used to calculate bacterial diversity, a higher Simpson index value indicates lower community diversity, while a higher Shannon index value indicates greater community diversity. The coverage index is a measure of how well the sequencing results represent the actual sample. It reflects whether the sequencing accurately captures the true composition of the sample. β diversity was calculated using Qiime [20], Student’s t-test was used to test the significance of differences between groups, and Spearman’s analysis was used to analyze the association between tumor markers and tumor microbiome.

### Statistical analysis

This study utilized Statistical Product and Service Solutions(SPSS) 25.0 software for conducting statistical analysis. For continuous measurement data that followed a normal distribution, statistical description was performed using the mean ± standard deviation, and the t-test was used for analyzing indicators with equal variances. In cases where the data did not conform to the normal distribution, the data was described using quartiles, and the *W*_*Mann−Whitney*_ test was used for analysis. The Chi-square test was employed for analyzing categorical data. A significance level of *P* < 0.05 was considered statistically significant.

## Results

### Basic information about fecal sample donors

A total of 38 feces samples were included in this study, including 9 from healthy controls and 29 from pancreatic cancer patients. The results indicated that the average age of pancreatic cancer patients was higher compared to that of healthy controls(*P* < 0.05). Additionally, there was no significant difference observed in BMI and gender between pancreatic cancer patients and healthy controls(*P*<0.05) (Table 1).

**Table 1:** Basic Information of Participants

Variable		Healthy control	Pancreatic cancer	*Statistics*	*P*-value
Age (year)		30.54 ± 6.75	67.55 ± 10.78	*t* = 10.142	0.000
BMI (kg/m^2^)		20.84 ± 1.46	22.40 ± 2.90	*t* = 1.622	0.113
Gender	male	3	17	χ^2^ = 0.893	0.345
	female	6	12		

Note. BMI, Body Mass Index = weight/height squared (kg/m^2^); *P* < 0.05 difference is statistically significant

### Abnormal changes in gut microbes in pancreatic cancer patients

**Fig. 1:**
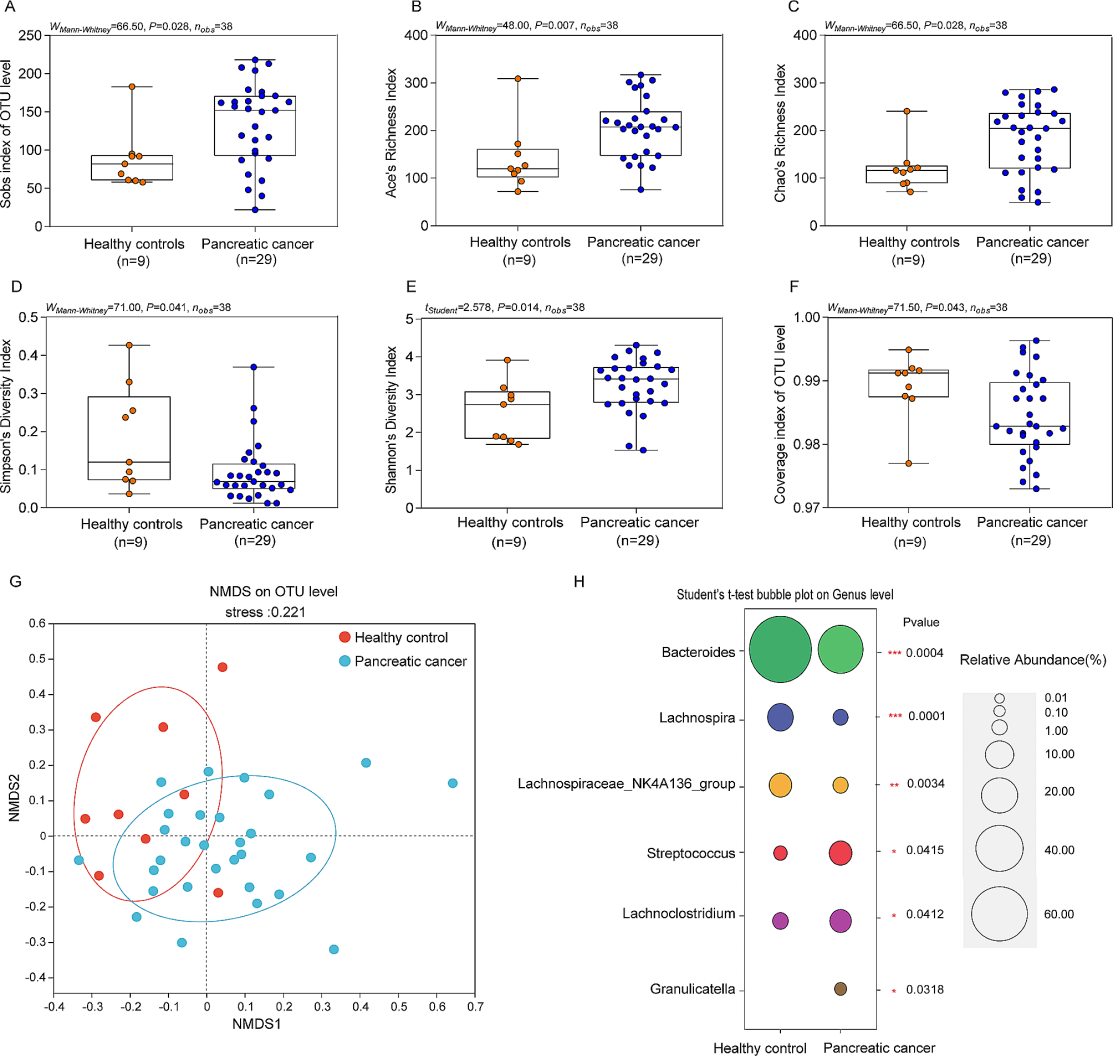
Comparison of gut microbes in patients with pancreatic cancer and healthy people. (**A**)Microbial richness based on the Sobs index; (**B**-**C**)Index of community richness; (**D**-**E**)Index of community diversity; (**F**)Microbial sequencing coverage index. (**G**)Non-metric multidimensional scaling (NMDS) is a multidimensional data down scaling method used to analyze similarities and differences between pancreatic cancer and healthy people. According to the NMDS Bray-Curtis similarity diagram, the two-dimensional stress value is 0.221. (**H**)Bubble plot showing differences in abundance of the six most abundant genus level in the gut microbiota of patients with pancreatic cancer and healthy humans.Different colors represent different bacterial groups, and the size of the circles indicates the relative abundance of each bacterial group. ^*^: *P* < 0.05; ^**^: *P* < 0.01; ^***^P: <0.001. (**A**-**D**, **F**)using a one-tailed Mann-Whitney test.(**E**, **H**)using an unpaired two-tailed Student’s t test

In 29 fecal samples, we detected a total of 104,082 valid sequences. After removing redundant sequences, the sequences are clustered by OTU with a similarity of 0.97. The Venn diagram results showed that the number of unique OTUs in pancreatic cancer was 38, the number of unique OTUs in healthy controls was 700, and the number of common OTUs in both groups was 338(Supplementary Fig. S1A). Analysis of alpha diversity results showed that the Sobs index, Ace index, Chao index and Shannon index of gut microorganisms in pancreatic cancer were higher than those in healthy controls(*P*<0.05)(Fig. 1A-D), while the Simpson index in pancreatic cancer was lower than that of healthy controls(*P*<0.05) (Fig. 1E). Convergence index showed that there was no difference in species converge between pancreatic cancer and healthy controls(*P<*0.05) (Fig. 1F). The above results indicate that pancreatic cancer can significantly increase the species richness and diversity of gut microbes in patients.

Non-metric multidimensional scaling (NMDS) is a multidimensional data down scaling method used to analyze similarities and differences between pancreatic cancer and healthy people. According to the NMDS Bray-Curtis similarity diagram, the two-dimensional stress value is 0.221 (Fig. 1G).

We further performed a species composition analysis of the gut microbes, at the genus level, the results showed that the dominant species of gut microbes in pancreatic cancer and healthy people are Bacteroides, Lachnospira, Lachnospiraceae_NK4A136_group(Supplementary Fig. S1C), at the same time, there were statistically significant differences in species abundance between the two groups(*P* < 0.05) (Fig. 1H).

At that specie level, the results showed that the species were mainly *Bacteroides_vulgatus*, *Bacteroides_plebeius*,*unclassified_g__Lachnospira*, *Lachnospiraceae_bacterium_GAM79* (*P* < 0.05)(Supplementary Fig. S1B). The above results indicated that pancreatic cancer significantly changed the species composition of intestinal microbiota.

### Basic information about diseased tissue sample donors and clinical data

This study included 28 tissue samples, including 14 patients with chronic pancreatitis and 14 patients with pancreatic cancer. There were no statistical differences between the two groups in terms of age, BMI, and gender composition(*P<*0.05). The expression levels of tumor markers CA242 and CA19-9 in the serum of patients with pancreatic cancer are higher than those of patients with chronic pancreatitis(*P* < 0.05)(Table 2).

**Table 2:** Basic information of study subjects and detection of tumor markers

Variable		Chronic pancreatitis	Pancreatic cancer	*Statistics*	*P*-value
Age (year)		52.35 ± 9.97	56 ± 7.09	*t* = 1.114	0.275
BMI(kg/m^2^)		21.53 ± 2.11	20.56 ± 3.83	*t* = 0.762	0.455
Gender	male	12	7	χ^2^ = 2.602	0.106
	female	2	7		
Tumor markers	CA125(U/L)	12.45(117.70–5.64)	18.82(52.39–10.32)	*W*_*Mann−Whitney*_=56.000	0.538
	CA242(IU/ml)	3.37(4.20–1.59)	23.23(38.70-11.65)	*W*_*Mann−Whitney*_=1.000	0.002
	CEA(ng/ml)	2.93(3.82–1.73)	2.49(3.60–1.61)	*W*_*Mann−Whitney*_*=*54.500	0.789
	CA19-9(U/L)	10.90(21.55–6.80)	274.92(466.58–72.93)	*W*_*Mann−Whitney*_*=*11.000	0.001

*Note* BMI, Body Mass Index = weight/height squared (kg/m^2^); CA125, carbohydrate antigen 125; CEA, carcinoembryonic antigen; CA242, carbohydrate antigen 242; CA199, carbohydrate antigen 199; *P*<0.05 difference is statistically significant

### Receiver operating characteristic(ROC) curve analysis of tumor markers

**Fig. 2:**
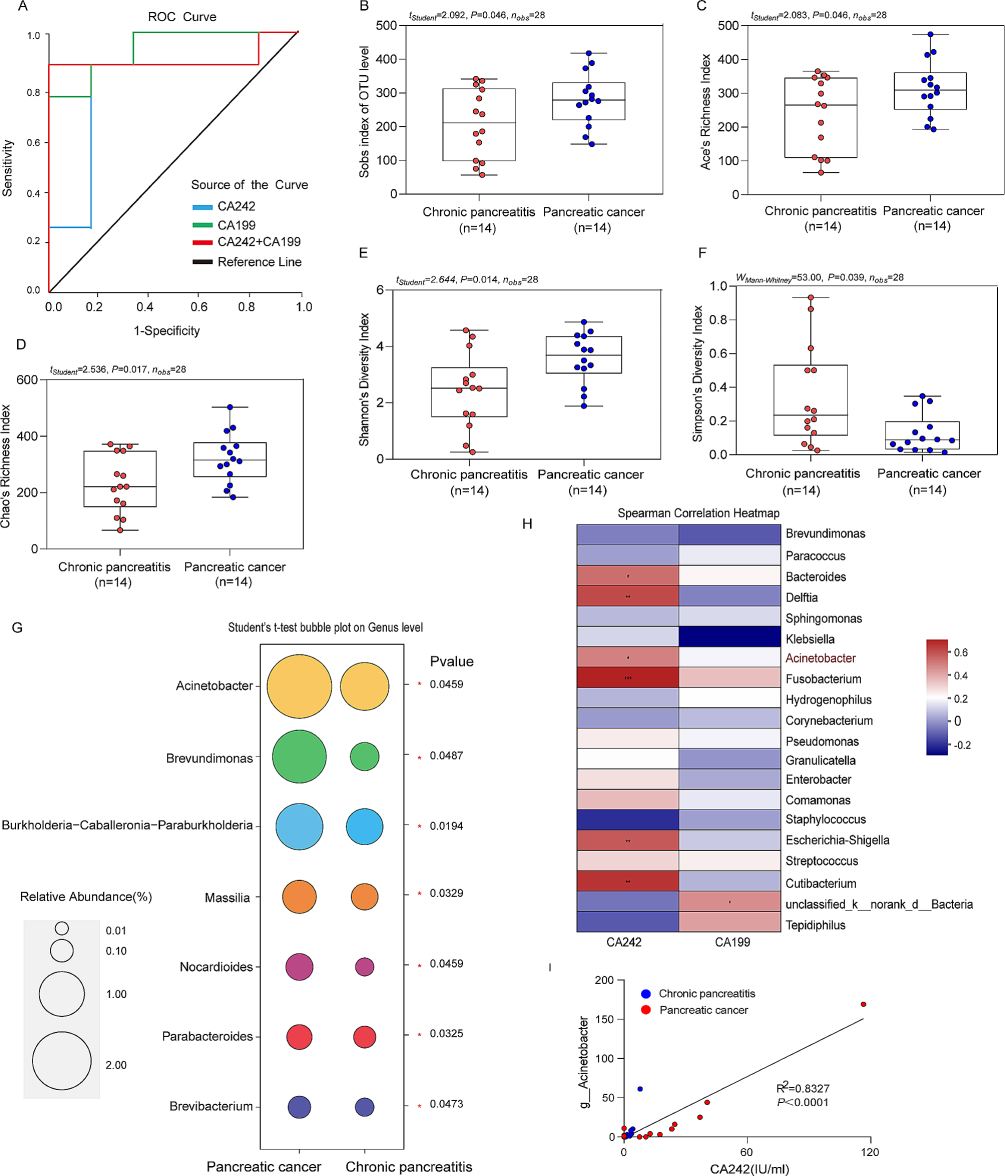
Tumor marker survival curves and microenvironmental changes in patients with pancreatic cancer. (**A**)Survival curve analysis of tumor markers(CA242 and CA199). (**B**)Microbial richness based on the Sobs index; (**C**-**D**)Index of community richness; (**E**-**F**)Index of community diversity. (**G**)Bubble plot showing differences in abundance of the seven most abundant genus level in the tumor microorganisms. of patients with pancreatic cancer and chronic pancreatitis. (**H**)Correlation analysis between tumor markers and microorganism, the mean relative abundance of the microbiome in CA242 and CA199 is represented by the color of the spots in the right panel. ^*^: *P* < 0.05;^**^: *P* < 0.01;^***^: *P* < 0.001. (**I**)Acinetobacter showed a significant positive correlation with CA242. (**B**-**E**, **G**)using an unpaired two-tailed Student’s t test. (**F**)using a one-tailed Mann-Whitney test

The diagnostic performance of serological markers in pancreatic cancer prediction is summarized and quantified by ROC curve analysis(Fig. 2A). In Table 3, the performance of various tumor markers is assessed using metrics such as the Area Under Curve (AUC), P-value, and 95% Confidence Interval(CI) bounds. The analyzed tumor markers include CA242, which exhibits an AUC of 0.854 (*P* = 0.028, 95% CI: 0.617-1.000), CA19-9 with an AUC of 0.938 (*P* = 0.007, 95%CI: 0.814-1.000), and the combination of CA242 and CA19-9 showing the highest AUC at 0.986 (*P* = 0.014, 95% CI: 0.698-1.000). These results indicate the diagnostic potential of these markers.

**Table 3:** ROC curve characteristics of different tumor markers

Tumor markers	AUC	*P*-value	95% Confidence Interval	
			Lower Bound	Upper Bound
CA242	0.854	0.028	0.617	1.000
CA199	0.938	0.007	0.814	1.000
CA242 + CA199	0.986	0.014	0.698	1.000

*Note* ROC, Receiver Operating Characteristic; AUC, Area Under Curve; *P*<0.05 difference is statistically significant

In Table 4, the optimal cutoff values for various tumor markers are detailed, along with associated metrics. CA242 is found to have an optimal cutoff value with a sensitivity of 0.875, CA19-9 exhibits an optimal cutoff value resulting in a sensitivity of 0.75. Similarly, the combination of CA242 and CA19-9 demonstrates an optimal cutoff value with a sensitivity of 0.875. These findings elucidate the diagnostic accuracy and potential of these tumor markers at their respective optimal cutoff values.

**Table 4:** Optimal cutoff values of ROC curves for different tumor markers

Tumor markers	*Sensitivity*	1-*Specificity*	*Youden’s index*	*Specificity*
CA242	0.875	0.167	0.708	0.833
CA199	0.75	0	0.75	1
CA242 + CA199	0.875	0	0.875	1

### Abnormal microbial alterations in tumors of pancreatic cancer patients

Alpha diversity analysis shows that patients with pancreatic cancer have higher Sobs index, Ace index, Chao index and Shannon index than patients with chronic pancreatitis(*P* < 0.05) (Fig. 2B-E). At the same time, the Simpson index in pancreatic cancer was lower than patients with chronic pancreatitis(*P* < 0.05) (Fig. 2F). There was no statistical difference in Coverage index between the two groups (*P*>0.05)(Supplementary Fig. S1D).The above results show that pancreatic cancer can significantly increase the species richness and diversity of microbiome in tumor.

According to the NMDS Bray-Curtis similarity diagram, the two-dimensional stress value is 0.139(Supplementary Fig. S1F). The results of NMDS analysis showed that the structure of the microbial community between samples from pancreatic cancer and chronic pancreatitis was highly similar.

At the family level, the results show that the differential species of microbiome in focal tissues in patients with pancreatic cancer and chronic pancreatitis are Moraxella, Sphingomonas and Oxalobacteriae(*P* < 0.05)(Supplementary Fig. S1F). At the genus level, the results showed that the differential species of microorganisms in focal tissues in patients with pancreatic cancer and chronic pancreatitis were Acinetobacter, Brevundimonas and Burkholderia-Caballeronia-Paraburkholderia(*P* < 0.05)(Fig. 2G). The above results indicated that pancreatic cancer could significantly change the species composition of microbiome in tumor.

### Correlation analysis between tumor microbiome and tumor markers

According to *Spearman* correlation analysis, expression levels of tumor marker CA242 were consistent with Bacteroides, Delftia, Acinetobacter, Fusobacterium, Escherichia/Shigella and propionibacterium(*P* < 0.05)(Fig. 2H). Further analysis, Acinetobacter showed a significant positive correlation with CA242(*P* < 0.05)(Fig. 2I). Based on the results of species difference and correlation analysis of microbiome in focal tissues of patients with chronic pancreatitis and pancreatic cancer, it was concluded that Acinetobacter is a specific bacterial genus highly expressed in pancreatic cancer tissue.

## Discussion

### Pancreatic cancer leads to increased expression of serum tumor markers in patients

Preoperative serum biomarker detection is one of the methods to improve the prognosis of pancreatic cancer. The results of this study showed that CA19-9 and CA242 levels were significantly elevated in patients with pancreatic cancer compared with patients with chronic pancreatitis.Increases in CA19-9 have been found to be associated with the onset and progression of pancreatic cancer [21]. However, it is important to note that this increase may only be observed when the tumor has reached a certain size [4]. Additionally, CA242 is a highly specific serum marker for pancreatic cancer, and research indicates that patients with positive CA242 results have a notably shorter survival time than those with negative results [22]. When considering the predictive capacity for Pancreatic cancer, these markers demonstrate considerable promise. In our study, CA242 and CA19-9 demonstrated specificities of 83.3% and 100%, respectively, for the diagnosis of pancreatic cancer. Previous studies have confirmed the excellent specificity of CA19-9 [23] and confirmed its usefulness as an independent marker for pancreatic cancer. CA242, while less studied, has shown promising specificity and could complement CA19-9 in diagnostic applications [24]. Of note, the use of a single tumor marker alone may have limited utility for disease screening and diagnosis. Therefore, the use of multiple markers can help avoid misdiagnosis and reduce unnecessary diagnosis and treatment burden on patients. In conclusion, our data support the use of CA242 and CA19-9 as reliable markers in the predictive diagnostics of pancreatic cancer.

### Pancreatic cancer causes gut microbiota disorders in patients

The results of this study suggest that pancreatic cancer may result in increased abundance and diversity of patient’s gut microbiota. However, some studies [25] have provided inconclusive results and demonstrated no significant differences in species diversity in colonic lavage fluid samples from pancreatic cancer patients and healthy controls. The possible reason for these inconsistent findings could be attributed to varying sample collection techniques [26]. The findings of this study indicated that patients with pancreatic cancer displayed a decrease in intestinal microbial primarily caused by Bacteroides, Lachnospira and Lachnospiraceae_NK4A136_group at the genus level. Relevant studies have reported that Lachnospiraceae significantly affects host health by producing short-chain fatty acids (SCFA) to promote colonization resistance against intestinal pathogens [27]. Additionally, research has demonstrated that an imbalance in intestinal flora in pancreatic cancer patients is marked by decreased species diversity, accompanied by a boost in the number of lipopolysaccharide -producing bacteria and a reduction in bacteria producing SCFA [28]. Lachnospira_NK4A136_group is deemed an anti-inflammatory factor and beneficial for intestinal health due to its production of SCFA [29]. Bacteroides are crucial symbiotic bacteria in the human intestinal tract that help break down food to produce nutrients and energy required by the body [30]. Furthermore, a cross-border study showed that Streptococcus increased in the intestines of patients with pancreatic cancer [31]. Therefore, microbial imbalance may contribute to the occurrence and development of pancreatic cancer because it disrupts the balance between the intestinal microbiota and the body, leads to the production of relevant inflammatory mediators, and activates immune functions and related signaling pathways through graded responses [32, 33]. This, in turn, induces DNA strand damage and a series of oxidative stress responses, ultimately leading to tumor formation [34]. Therefore, the gut microbiota may indirectly intervene in pancreatic cancer by participating in mediating inflammatory and immune responses.

### Changes of microbial composition in tumor microenvironment caused by pancreatic cancer

Although the pancreas has long been considered a sterile organ, recent research into the mechanisms of pancreatic disease has revealed new evidence for the presence of microorganisms in both normal and diseased pancreas [35, 36]. It is generally accepted that microorganisms can migrate retrogradely through the duodenum to the pancreas or invade through the mesenteric veins and lymphatic system due to intestinal permeability impairment [37, 38]. These microorganisms have been found to have a significant impact on the occurrence, development, and treatment of diseases [13, 39]. This study discovered that microbial communities in pancreatic cancer tissues exhibit a greater diversity and abundance when contrasted with those found in tissues with chronic pancreatitis. Similar results were found in related studies [40].

The results of this study showed that the species abundance of Acinetobacter was significantly increased at the genus level in pancreatic cancer tissue samples compared with chronic pancreatitis tissues. Most recently, metagenomic sequencing was utilized to comprehensively analyze the microbial population of pancreatic tumors, revealing that *Acinetobacter, Pseudomonas,* and *Sphingosine* may be significantly implicated in carcinogenesis [41]. Acinetobacter exhibits complex and extensive drug resistance, which can rapidly evade the virulence of the innate immune system, reach higher bacterial density and trigger severe inflammatory responses [42], thereby affecting the expression levels of tumor markers. Moraxella, a common conditional pathogen, belongs to *Acinetobacter* at the genus level. In a separate study involving patients with pancreatic cancer in China, it was observed that the presence of *Moraxella* was markedly elevated in tongue coating and saliva samples [43, 44]. Nejman et al. analyzed the microbiome of the tumor and adjacent normal tissue and showed that Proteobacteria play a dominant role in the microbiome of pancreatic cancer [45]. Further, Geller et al. conducted research that observed the enrichment of γ-*proteobacteria* in pancreatic cancer upon comparison of bacterial DNA in pancreatic cancer tissue with that in normal tissue [13].

Based on the results of species difference and correlation analysis of microbiome in focal tissues of patients with chronic pancreatitis and pancreatic cancer, it was hypothesized that *Acinetobacter* might be the marker genus of Pancreatic Cancer. The potential for quantitative correlation between these markers and specific microbial populations, which have been increasingly implicated in pancreatic carcinogenesis [46]. An integrated approach that combines traditional tumor markers with novel molecular markers influenced by specific microbial signatures may result in a versatile diagnostic tool that improves early detection and personalized treatment strategies for pancreatic cancer.

There are some limitations to this study. Firstly, the sample size of this study was small. Secondly, the presence of an age imbalance in the healthy control group may potentially undermine the true validity of the results obtained. To enhance the reliability of future studies, it is recommended to increase the sample size and ensure a balanced classification. Thirdly, only studying serum markers to identify the risk of pancreatic cancer patients did not evaluate the validity of ROC curve characteristics. Fourthly, future prospective studies should incorporate more clinical factors, especially factors highly related to pancreatic cancer, to further evaluate the effectiveness of microbiome combined with tumor markers in predicting the occurrence of pancreatic cancer.

## Conclusion

In summary, our study found that the expression levels of serum tumor markers CA242 and CA19-9 were increased in patients with pancreatic cancer. In patients with pancreatic cancer, beneficial bacteria in the intestinal tract and tumor microenvironment decrease, while pathogenic bacteria increase, leading to a deterioration of the microecology. Acinetobacter is a specific bacterial genus highly expressed in pancreatic cancer tissues.

### Electronic supplementary material

Below is the link to the electronic supplementary material.

Supplementary Material 1

## Data Availability

The sequences analyzed during this study have been deposited in Genbank repository under the accession number PRJNA 1029515.

## Abbreviations

PC: Pancreatic Cancer
CA19-9: Carbohydrate Antigen 19-9
CA242: Carbohydrate Antigen 242
DNA: Deoxyribonucleic Acid
PCR: Polymerase Chain Reaction
OTU: Operational Taxonomic Unit
NMDS: Non-metric Multidimensional Scaling
Sobs: Species Observation
Ace: Abundance-based Coverage Estimator
SPSS: Statistical Product and Service Solutions
NMDS: Non-metric Multidimensional Scaling
BMI: Body Mass Index
CEA: Carcinoembryonic Antigen
CA125: Carbohydrate Antigen 125
ROC: Receiver Operating Characteristic
CI: Confidence Interval
AUC: Area Under the Curve
