# Supplementary material for: Heterogeneous changes in gut and tumor microbiota in patients with pancreatic cancer: insights from clinical evidence
Source: BMC Cancer. 2024 Apr 15;24:478. doi: 10.1186/s12885-024-12202-z (PMC11020926; doi:10.1186/s12885-024-12202-z)
Supplement: Supplementary file 1 — Supplementary Material 1 [file 12885_2024_12202_MOESM1_ESM.docx]

**Figure Legends**


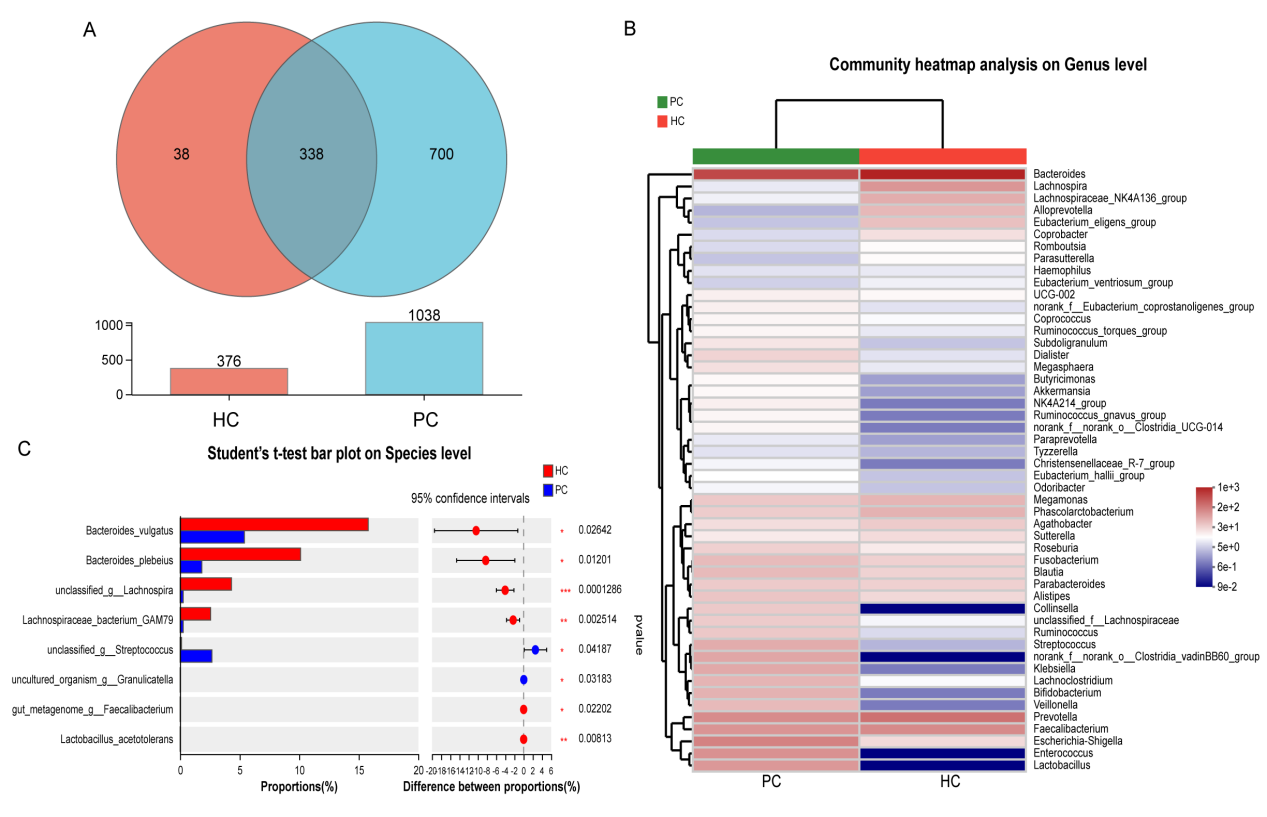
**Supplementary Figure 1 Comparison of gut microbes between pancreatic cancer and healthy**

Comparison of gut microbes between pancreatic cancer and healthy.(A)Venn diagram of OTUs between HC and PC groups;(B)In the Heatmap diagram, the abundance difference of different bacteria in PC and HC is represented by the color depth. (C)The darker the color, the higher the abundance of modified species.Data of PC and HC groups were showed as relative abundance (%) of species . **P* <0.05, ***P* <0.001,****P* <0.0001


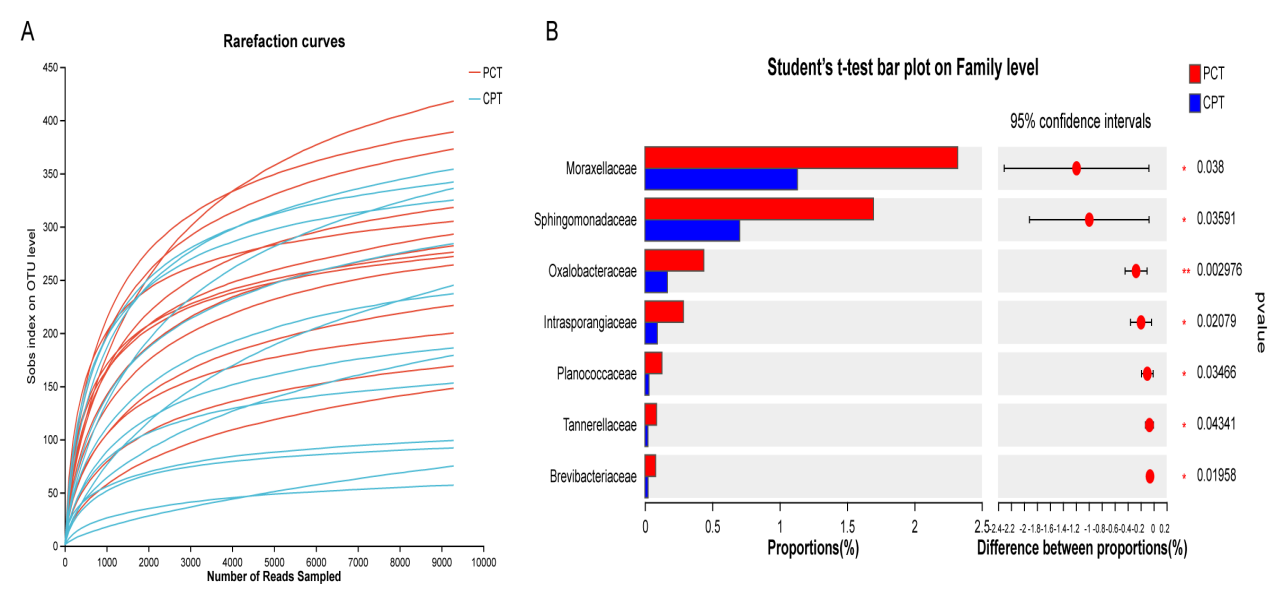
**Supplementary Figure 2 Changes of microbial composition in tumor microenvironment caused by pancreatic cancer**

(A) The rarefaction curves are based on the amount of data drawn from CPT and PCT samples as the x-axis and the Sobs index values as the y-axis. (B) Data of CPT and PCT groups were showed as relative abundance (%) of family. **P* <0.05, ***P* <0.001
